# Hemispheric Asymmetry of Endogenous Neural Oscillations in Young Children: Implications for Hearing Speech In Noise

**DOI:** 10.1038/srep19737

**Published:** 2016-01-25

**Authors:** Elaine C. Thompson, Kali Woodruff Carr, Travis White-Schwoch, Adam Tierney, Trent Nicol, Nina Kraus

**Affiliations:** 1 Auditory Neuroscience Laboratory, Northwestern University, Evanston, Illinois 60208, USA; 2 Department of Communication Sciences, Northwestern University, Evanston, Illinois 60208, USA; 3 Institute for Neuroscience, Northwestern University, Evanston, Illinois 60208, USA; 4 Department of Neurobiology & Physiology, Northwestern University, Evanston, Illinois 60208, USA; 5 Department of Otolaryngology, Northwestern University, Evanston, Illinois 60208, USA.

## Abstract

Speech signals contain information in hierarchical time scales, ranging from short-duration (e.g., phonemes) to long-duration cues (e.g., syllables, prosody). A theoretical framework to understand how the brain processes this hierarchy suggests that hemispheric lateralization enables specialized tracking of acoustic cues at different time scales, with the left and right hemispheres sampling at short (25 ms; 40 Hz) and long (200 ms; 5 Hz) periods, respectively. In adults, both speech-evoked and endogenous cortical rhythms are asymmetrical: low-frequency rhythms predominate in right auditory cortex, and high-frequency rhythms in left auditory cortex. It is unknown, however, whether endogenous resting state oscillations are similarly lateralized in children. We investigated cortical oscillations in children (3–5 years; N = 65) at rest and tested our hypotheses that this temporal asymmetry is evident early in life and facilitates recognition of speech in noise. We found a systematic pattern of increasing leftward asymmetry for higher frequency oscillations; this pattern was more pronounced in children who better perceived words in noise. The observed connection between left-biased cortical oscillations in phoneme-relevant frequencies and speech-in-noise perception suggests hemispheric specialization of endogenous oscillatory activity may support speech processing in challenging listening environments, and that this infrastructure is present during early childhood.

The left and right auditory cortices are specialized to process acoustic features at different time scales, such as those conveyed by speech, simultaneously. Converging evidence suggests that slow, low-frequency temporal features (~200 ms; 3–7 Hz; syllables) are biased to right hemisphere auditory cortex, whereas fast, high-frequency temporal features (~20–50 ms; 20–50 Hz; phonemes) are biased leftward^1^,^2^,^3^,^4^. Parsing of a speech stream into finer units of information is hypothesized to occur through phase-locking of left-lateralized gamma oscillations and right-lateralized theta oscillations^5^,^6^,^7^. This notion, initially put forth by Poeppel in the Asymmetric Sampling in Time (AST) hypothesis^7^, suggests asymmetry of hemispheric neural oscillations provides biological scaffolding to facilitate speech perception, and solves the problem of simultaneously processing acoustic information across timescales.

Consistent with the predictions of the AST hypothesis, recent investigations report endogenous (i.e. resting state) neural ensembles in the auditory cortices sample at rates that are tuned to fundamental speech units, and exhibit a similar asymmetry as speech-evoked activity. For instance, a simultaneous functional magnetic resonance imaging and electroencephalography experiment in adults revealed endogenous left auditory cortex activity correlated with high-frequency resting-state oscillations, whereas endogenous right auditory cortical activity correlated to low-frequency resting oscillations^8^. Direct recordings from human cortex also show high-frequency oscillations predominate in the left hemisphere at rest, while low-frequency oscillations predominate in the right^9^. Taken together, these results demonstrate an asymmetric pattern of resting state neural oscillations in adults. The similarity of this activity with speech-evoked phaselocking leads to the hypothesis that these resting-state asymmetries play a role in speech perception^9^.

Speech perception is important for communication, but is compromised by challenges in everyday listening conditions such as background noise and reverberation. Our hypothesis is that asymmetric sampling facilitates speech understanding in challenging listening environments. While speech-evoked hemispheric oscillatory asymmetry has been observed in infants, young children, and adults, the functional consequences of *resting* asymmetry for speech understanding have only been hinted at in adults^8^,^9^,^10^, and remain unknown during early childhood. This is a particularly critical question with respect to child development, because young children are more vulnerable to deleterious effects of noise than older children and adults^11^.

We collected resting-state cortical activity in young children, whom we also tested on a word perception in noise task, to investigate two main questions: first, is the hemispheric distribution of endogenous neural oscillations asymmetric during early childhood? And if so, does this asymmetry facilitate speech-in-noise perception? We predicted children who were better at perceiving speech in noise would have greater asymmetrical oscillatory activity at rest, with high-frequency oscillations lateralized to the left hemisphere and low-frequency oscillations to the right.

## Results

### Endogenous neural oscillations are asymmetric in children

We measured resting oscillatory activity by asking children to sit quietly with eyes open while wearing an electrode cap for three minutes. We determined the amount of oscillatory activity from 2–55 Hz across the scalp, and analyzed the extent of lateralized activity by comparing activity between corresponding right and left electrodes.

**Figure 1:**
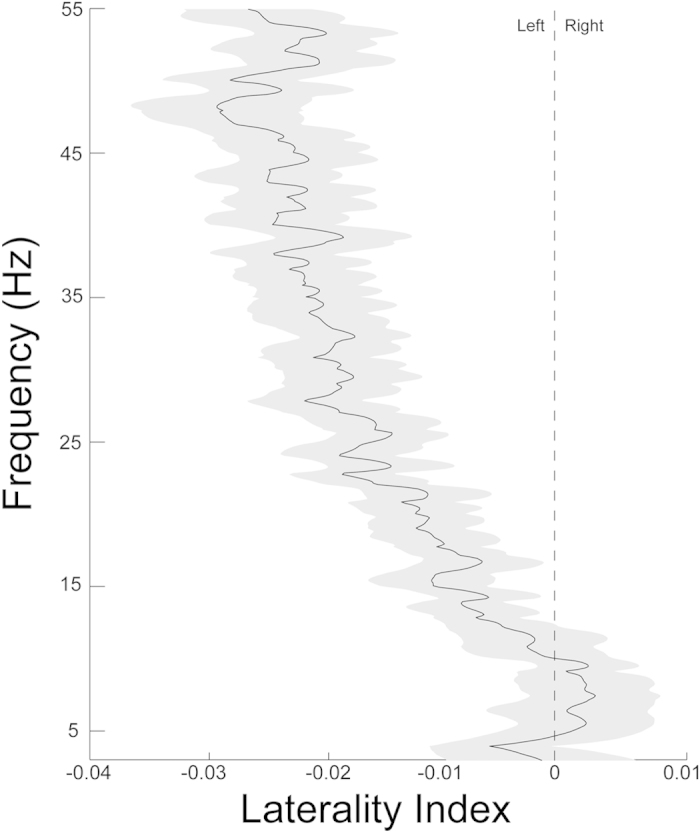
High-frequency resting oscillations are left-lateralized in children. Linear regression of spectral power reveals cerebral asymmetry at high (20-50 Hz) but not low (3-7 Hz) frequencies. The extent of left asymmetry increases with increasing frequencies into the gamma range. The shaded region represents one standard error.

High-frequency (HF) oscillations were more left-lateralized than low-frequency (LF) oscillations (Fig. 1; t(64) = 5.069, p < 0.001, LF laterality index mean = −0.002, HF laterality index mean = 0.0154; Cohen’s d = 0.682). In addition, HF oscillations were reliably lateralized (one-sample t-test comparing laterality index for HF from zero; t(64) = 4.557, p < 0.001; Cohen’s d = 1.139), whereas LF oscillations were symmetrically distributed across hemispheres (t(64) = −0.692, p > 0.05; Cohen’s d = 0.173).

Next, we asked if these patterns of oscillatory asymmetry change between the ages of 3 and 5, and if they are distinct in males and females. There were no relations between age and the LF or HF asymmetry indices, suggesting these patterns of asymmetry do not mature during this period of early childhood (HF: *ρ*(64) = −0.231, p = 0.064; LF: *ρ*(64) = −0.126, p = 0.319). We determined the slope of each child’s asymmetry pattern (extent of leftward laterality for increasing frequencies) to obtain a global measure of oscillatory asymmetry; the slope did not relate to age (*ρ*(64) = −0.171, p = 0.172), further suggesting oscillatory asymmetry does not mature during this age range. Finally, we found that males and females did not differ on any measures of oscillatory asymmetry (slope: F(1,64) = 0.007, p = 0.932; HF asymmetry index: F(1,64) = 1.713, p = 0.195; LF asymmetry index: F(1,64) = 0.139, p = 0.711). To confirm that HF oscillations are more left-lateralized than LF oscillations regardless of age or sex, we compared laterality index between HF and LF bands controlling for sex; although the HF oscillations were more left-lateralized than the LF oscillations (F(1,64) = 4.237, p = 0.044) the repeated measures ANCOVA showed no effects of age (F(1,64) = 2.947, p = 0.091), sex (F(1,64) = 0.872, p = 0.354), or interactions (all p > 0.3).

### Children with stronger high-frequency asymmetry hear better in noise

Speech-in-noise perception related to age, with older children performing better than their younger peers (r(59) = −0.353, p = 0.006), and sex, with males outperforming females (F(1,58) = 6.55, p = 0.013).

**Figure 2:**
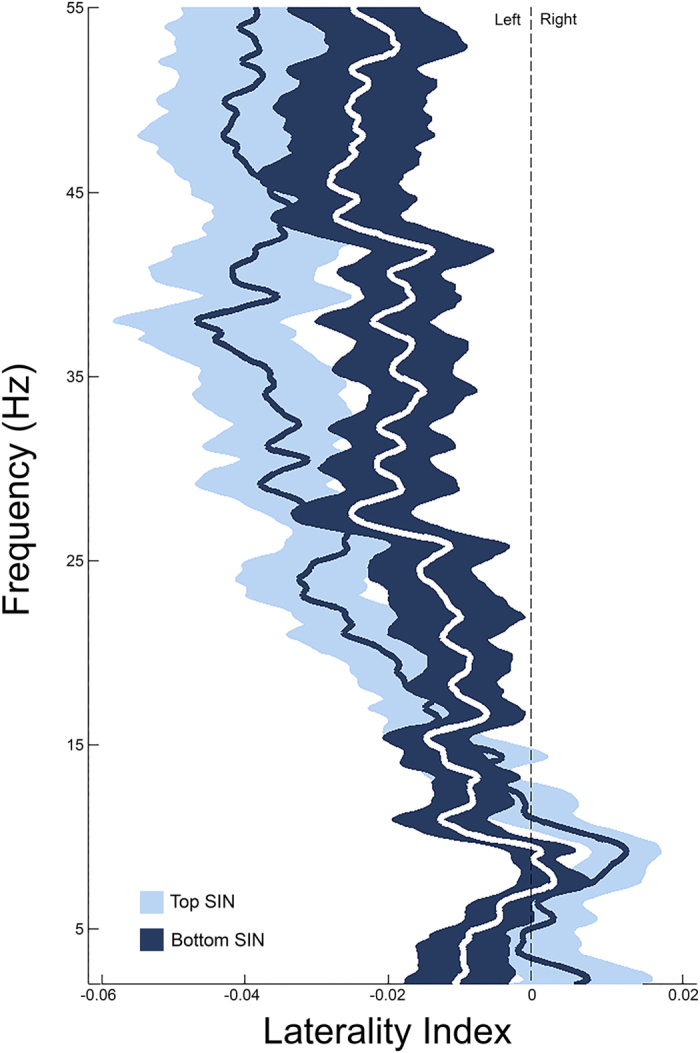
Children who are better perceivers of speech in noise have stronger left-lateralization of high-frequency endogenous oscillations.

**Figure 3:**
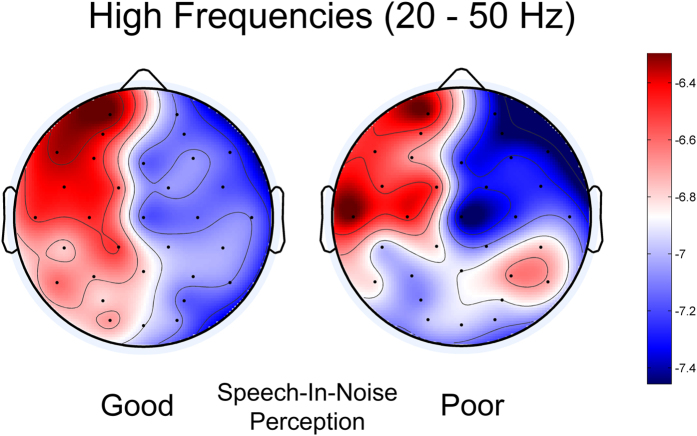
High-frequency cortical oscillatory activity was more left lateralized for children who were better perceivers of speech in noise. Topographic plots show the distribution of high-frequency (20–50 Hz) oscillations in good (left) and poor (right) perceivers of speech in noise. Red indicates more spectral power, and a leftwards bias for high-frequency oscillations is evident, particularly in the good speech-in-noise perception group.

Next, we investigated a potential relation between speech-in-noise perception and oscillatory asymmetry. We measured children’s ability to understand words in noise and grouped children as “Good”, “Average”, or “Poor” perceivers (tercile split, see Table 1 for demographics). Children with larger asymmetry slopes (i.e., stronger patterns of relative leftward asymmetry for higher-frequency oscillations) performed better on the speech-in-noise test (larger slope correlated to lower speech reception thresholds, controlling for age and sex, r(56) = −0.341, p = 0.009). Group comparisons showed that HF cortical oscillatory activity was the most left-lateralized in the “Good” perceiver group (group x frequency interaction; F(2, 54) = 5.932, p = 0.005*; η*^*2*^_*p*_ = 0.18; Figs 2 and 3).

## Discussion

Our results are twofold. First, we show that hemispheric asymmetry of resting oscillations is present in early childhood: resting-state high-frequency (20–50 Hz) cortical rhythms are biased to the left, yet low-frequency (3–7 Hz) cortical rhythms are balanced between the left and right hemispheres. Second, greater left-lateralization of high-frequency cortical oscillations is associated with better speech-in-noise perception. Overall, these results are consistent with predictions of the AST hypothesis, and align with converging evidence investigating evoked hemispheric oscillatory differences using electrophysiology^4^,^12^,^13^,^14^, functional neuroimaging^2^,^15^ and optical tomography^16^. Importantly, we show that this hypothesis predicts high-frequency oscillatory patterns during early childhood, and that individual differences in the extent of HF asymmetry relate to speech-in-noise perception. In contrast to the predictions of AST, however, low-frequency oscillations were *symmetrically* distributed. These results suggest that cortical rhythms at phonemic rates, but not slower rates, are lateralized during early childhood.

The absence of a low-frequency bias could be due to a number of factors. One possibility is that right hemisphere auditory cortex develops more slowly than left auditory cortex. Developmental studies reveal frontotemporal white matter tracts in the left hemisphere, but not the right, are mature by late childhood^17^. Additionally, structural changes in right, but not left, temporal cortical grey matter persist through adolesence^18^,^19^. Thus, an anatomical explanation for our results is that right hemisphere cortex is immature in our participants, leading to the prediction that as they mature low-frequency oscillations will become right-lateralized.

More often than not, speech understanding is compromised by the presence of background talkers, reverberation, and environmental noise. These challenges are particularly salient for young children. Learning in a classroom hinges upon precise speech perception and comprehension, but learning environments tend to be noisy^20^. A chief factor underlying successful comprehension is phase locking of neural oscillations to precisely extract a signal and suppress the noise. Here, we show that greater asymmetry of cortical rhythms *at rest* is linked to better recognition of words in noise. We speculate that a greater degree of oscillatory asymmetry at rest primes the brain to better perceive speech, particularly when challenged by a noisy background. Consistent with this idea is the finding that frontal high-frequency (gamma) oscillatory activity at rest is linked to the development of cognitive and language skills^21^,^22^, suggesting high-frequency oscillations may play a vital role in classroom learning. If endogenous oscillatory asymmetry primes the brain for speech perception, children with reduced or reversed asymmetry may be less equipped to perceive speech, especially in noise.

Indeed, perception of speech in background noise is a complex and multifaceted process that draws on cognitive mechanisms (memory, attention), linguistic and pragmatic processes (what was said and in what context), and sensory precision (encoding of the speech stream)^23^,^24^. Though each of these aspects support speech-in-noise perception in young childhood, our words in noise task may be considered more similar to a signal detection task due to its small cognitive and linguistic load^25^. We anticipate the relationship we found between resting oscillatory asymmetry and speech-in-noise perception is due to signal detection constraints rather than cognitive mechanisms or linguistic perception. Therefore, we predict a similar biological relationship with detection of all signals in noise, including pure tones; our finding likely does not rely upon the cognitive and linguistic mechanisms that operate during speech perception, but future work is needed to address this question.

While this work provides new insights into patterns of endogenous oscillatory asymmetry in children, a few limitations remain. First, the epoch size (1-second non-overlapping epochs) used for these analyses may not be ideal for low-frequency oscillations—because these oscillations are so low frequency, fewer than five cycles of each fits into this window size, potentially decreasing the resolution of our measurements. We raise this caveat for future work considering low-frequency endogenous oscillations. A second limitation is the scope of our speech perception in noise task: everyday speech perception is a function of complex interactions between talker, listener, and noise, and recognizing isolated words in babble may not reflect how these children perform in real-world listening conditions. This limitation dovetails with the broader challenge of testing young children; although we had good compliance on all of our measures, children this young still perform variably on these tests. Future work exploring links between asymmetry and a variety of speech perception tasks will be valuable to support such generalizations. A third limitation is the spatial specificity of our electroencephalography (EEG) measurements. Although EEG allows for precise temporal specificity of the oscillatory patterns, future research should investigate hemispheric differences using methodology that allows for more precise spatial resolution to confirm the hypothesis that these oscillatory asymmetries are a reflection of activity in auditory areas.

In summary, resting-state high-frequency oscillations are biased to the left hemisphere in young childhood, and the extent of this high-frequency asymmetry relates to the perception of speech in noise. Our results align with models of cortical asymmetry and speech perception, and illustrate a potential role for endogenous rhythms in priming the brain for perception of speech in background noise during early childhood.

## Methods

### Participant Demographics

Sixty-five children, ages 3.0–4.9 years old participated (M = 4.07, SD = 0.62). All children passed a hearing screening (Type A tympanograms and distortion product otoacoustic emissions > 6 dB above the noise floor from 0.5–4 kHz). No participant had a family history or diagnosis of a language-based learning disability. All children had normal scaled scores (within +/− 1 SD of mean) on verbal and nonverbal intelligence tests (Wechsler Preschool and Primary Scale of Intelligence; WPPSI^26^;). Written consent and informed assent were obtained from the parents and children, respectively. All procedures were approved by the Northwestern University Institutional Review Board. All procedures were carried out in accordance with the approved guidelines.

### Endogenous Neural Oscillatory Activity Acquisition & Analysis

Continuous electroencephalography (EEG) was recorded in a soundproof booth, lights on, while children rested in silence for 3 minutes with their eyes open. EEG was acquired using a 32-channel electrode cap (BioSEMI Active2 recording system) at a sampling rate of 512 Hz with an online band pass filter from 0.1–104 Hz. Electro-oculogram activity was monitored using two electrodes placed on the superior and outer canthi of the left eye. The online reference electrode was at CPZ per the hardware configuration of the BioSEMI electrode cap. Offsets were kept below 40 mV.

All data processing was performed offline in MATLAB. The continuous EEG was referenced to an average of the left and right earlobes, and manual artifact rejection was performed to eliminate segments contaminated by eyeblinks, muscle movements, and other artifact. Data were re-referenced to a common average (a digital average of all electrodes included in analyses). Cleaned recordings were then segmented into sixty 1-second non-overlapping epochs. The frequency spectrum of each epoch was then measured using a fast Fourier transform (5120 points), and the resulting frequency spectra were averaged and log transformed to create an average frequency spectrum for each channel.

### Speech Perception in Noise

The Children’s Realistic Index of Speech Perception (CRISP^27^) was used to determine speech reception thresholds (SRTs), which are defined as the sound level required to perceive a target word within competing speakers. CRISP employs an adaptive tracking procedure algorithm to calculate the SRT; by fixing the level of the competitor (female, conversational speech) to 55 dB SPL and varying the level of the target (male) based on a three-down/one-up procedure, thresholds were estimated based on the average of the final two reversals. Children were familiarized with age-appropriate words; to avoid potential confounds of vocabulary size, only the words correctly identified were used. An SRT of 55 dB SPL is equivalent to 0 SNR. The children were presented with a four-alternative forced choice paradigm, with four pictures representing possible responses, and instructed to point to the picture corresponding to the word they heard. Four children could not complete the task and were excluded from the speech perception analyses.

### Statistical Analyses

To calculate asymmetry, channels were divided into two groups: left (FP1, AF3, F3, FC1, FC5, C3, T7, CP1, CP5, P3, P7, PO3, O1) and right (FP2, AF4, F4, FC2, FC6, C4, T8, CP2, CP6, P4, P8, PO4, O2). Spectral EEG power was averaged across electrodes within each electrode grouping, and an “asymmetry index” was calculated with the following formula: (Right − Left)/(Right + Left). A number less than zero indicates a bias towards the left hemisphere.

Spectral energy was binned into frequency bands identified by Giraud and colleagues (2007): low (3–7 Hz, corresponding to theta oscillations) and high (20–50 Hz, corresponding to “low gamma” oscillations)^8^. To derive a more global measure of asymmetry across frequencies, a linear regression was performed for spectral energy between 2 and 55 Hz, resulting in a measure of slope (change in laterality index per unit frequency). A larger slope indicates a trend for leftward asymmetry to increase as frequencies increase.

Three t-tests were performed to determine the asymmetry index (within subjects factor: frequency range), testing differences in asymmetry between high and low frequencies (paired t-test), high frequencies and zero (one-sample t-test), and low frequencies and zero (one-sample t-test). A 2 × 3 repeated measures analysis of covariance (ANCOVA) (within subjects: high and low frequencies; between subjects: good, average, or poor words-in-noise group; covariates: age and sex) was performed to determine the relationship between asymmetry of cortical oscillatory activity and words-in-noise performance. Due to the potential influence of age, partial correlations (controlling for age) were performed to assess the relationship between asymmetry of cortical oscillatory activity and words-in-noise performance.

## Additional Information

**How to cite this article**: Thompson, E. C. *et al.* Hemispheric Asymmetry of Endogenous Neural Oscillations in Young Children: Implications for Hearing Speech In Noise. *Sci. Rep.* **6**, 19737; doi: 10.1038/srep19737 (2016).

## Figures and Tables

**Table 1:** A tercile split of speech-in-noise perception performance grouped the children into “Good”, “Average”, and “Poor” perceivers.

Speech-in-noise perception Group	Age (months, with SD)
Good *(n* = *20, 6F)*	50.2 (8.5)
Average *(n* = *21, 11F)*	50.81 (6.2)
Poor *(n* = *18, 13F)*	44.94 (6.4)
